# Effect of long term application of super absorbent polymer on soil structure, soil enzyme activity, photosynthetic characteristics, water and nitrogen use of winter wheat

**DOI:** 10.3389/fpls.2022.998494

**Published:** 2022-12-08

**Authors:** Yonghui Yang, Sensen Zhang, Jicheng Wu, Cuimin Gao, Defu Lu, Darrell W. S. Tang

**Affiliations:** ^1^ Institute of Plant Nutrition & Resource Environment, Henan Academy of Agricultural Sciences, Zhengzhou, China; ^2^ Yuanyang Experimental Station of Crop Water Use, Ministry of Agriculture, Yuanyang, China; ^3^ Field Scientific Observation and Research Station of Water Saving Agriculture in the Yellow River Basin of Henan Province, Yuanyang, China; ^4^ Henan Provincial Institute of Geology, Zhengzhou, China; ^5^ Geological Term of Henan Province Nonferrous Metals Geological Mineral Resources Bureaus, Zhengzhou, China; ^6^ Soil Physics and Land Management Group, Wageningen University & Research, Wageningen, Netherlands

**Keywords:** winter wheat, super absorbent polymer (SAP), soil microbial biomass carbon, soil enzyme activity, photosynthetic characteristics, water use, nitrogen use

## Abstract

**Introduction:**

Water scarcity and seasonal drought are major constraints on agricultural development globally. Super absorbent polymer (SAP) is a good amendment that can improve soil structure, increase soil water retention, and promote crop growth even with less soil moisture. We hypothesize that long term application of SAP has a better effect on soil organic carbon, soil enzyme activity, photosynthetic characteristics, yield, and water and nitrogen use than short term application.

**Methods:**

A long term field experiment with different application rates (0 (CK), 15 (L), 30 (M), 45 (H) kg ha^-1^) of SAP was conducted at the Yuzhou water conservation agriculture base of the Henan Academy of Agricultural Sciences from 2011 to 2019.

**Results and Discussion:**

The results indicate that applying SAP increases > 0.25 mm aggregates and decreased<0.25 mm aggregates in the soil after one year (2011) and 9 years (2019) of application. In addition, soil organic carbon, soil microbial biomass carbon, soil sucrase and cellulase activities, soil water consumption, water consumption, net photosynthetic rate (Pn), leaf water use efficiency (LWUE) of wheat and yield, all increased after SAP application. SAP also boosts water use efficiency and nitrogen use efficiency. Correlation analyses show that SAP promotes the growth of wheat, and improves the utilization rate of soil water and nutrients by improving the soil structure and increasing soil organic carbon and microbial enzyme activity.

**Conclusion:**

Based on our research, SAP treatment at a dosage of 45 kg ha^-1^ is most effective and is thus recommended.

## 1 Introduction

Drought and water shortage is an important limiting factor for the sustainable development of agriculture in China, especially in the arid area of the shallow hills in the north of China, where precipitation subject to seasonal drought is the main source of water for crops. Methods to improve the usage efficiency of limited precipitation and soil water resources, and how to improve soil water retention during periods of water shortage, are key issues in improving the water use efficiency in this area.

SAP is a polymer (Yang et al., 2005b), which can improve the structure of the soil (Terry & Nelson, 1986; Sojka et al., 2007), and promote the formation of aggregates (Yang et al., 2009a; Yang et al., 2021). SAP is known to be a “water reservoir” (Dorraji et al., 2015), and can absorb hundreds or even thousands of times its mass of water when sufficient water is present (Sojka and Entry, 2000; Dong et al., 2004; Ekebafe et al., 2011), reduce soil water loss (Al-Omran and Al-Harbi, 1997) and rapidly provide water for crops during periods of drought and water shortage (Arbona et al., 2005). Therefore, the application of SAP is an important measure in agricultural water conservation (Niu et al., 2007), and also a hot topic in the study of dry farming area (Huang et al., 1999). Studies show that the application of SAP could improve the root activity (Yang et al., 2011a) and physiological characteristics of wheat (Yang et al., 2011b), improve the water retention (Khadem et al., 2010) and water supply capability of soil (Song et al., 2009; Yang et al., 2012; Zhou et al., 2012), mitigate drought stress (Hüttermann et al., 2009; Yun et al., 2017), regulate soil porosity (Yang et al., 2011c; Yang et al., 2018; Yang et al., 2022) and the structural characteristics of soil aggregates (Wu et al., 2006; Yang et al., 2009a; Yang et al., 2021), enhance soil microbial activity (Dehkordi, 2018), stimulate the growth and development of crops (Yang et al., 2011d), and enhance photosynthesis (Yang et al., 2009b; Yang et al., 2011b), so as to achieve higher crop yields (Wu et al., 2007; Wu et al., 2010; Wu et al., 2011), precipitation and water use efficiency (Yang et al., 2011d; Lei et al., 2014), and nutrient utilization efficiency (Lents, 1998). Li et al. (2014) found that the application of SAPs did not lead to detectable adverse effects on the soil microbial community and might even enhance soil microbial activity. However, excessive water retention as a result of SAP application may have adverse effects on the soil, such as soil compaction (Pramthawee et al., 2017), increased bulk capacity, and reduced water transmission capacity (Yang et al., 2005a), which reduces microbial activity and soil enzyme activity, resulting in decreased soil quality and agricultural yields. In addition, at present, research on the effect of SAP on crop yield increase is mostly focused on short-term research, whereas systematic studies on soil organic carbon content, soil microbial biomass carbon, microbe enzyme, crop yield and water use efficiency after long-term application of SAP is rarely reported. Even less research has looked into the microbial biomass carbon and enzyme activity after application of SAP, especially in the long term.

We hypothesize that long-term application of SAP can improve soil structure, increase soil organic carbon content, soil enzyme activity, photosynthetic physiological properties, which then would result in an increase in crop yield and water use efficiency. This study is based on 9 years of results from a long-term experiment, and studies the influence of different rates of SAP application on soil structure, soil organic carbon content, soil microbial biomass carbon, sucrase activity and cellulase activity in 2011 and 2019, photosynthetic physiological properties from 2015 to 2019 and crop water use efficiency, nitrogen use efficiency and yield from 2011 to 2019. We aim to investigate the influence of the long-term application of such a water-retention enhancing substance on soil structure, organic carbon and microbial enzyme activity, photosynthetic physiological properties and whether it promotes improvements in crop water use, nitrogen use efficiency and crop yield. This would further our understanding of how the long-term application of SAP facilitates water conservation, and establish a new method of increasing water resource usage efficiency and agricultural production efficiency in dry farming areas.

## 2 Materials and methods

### 2.1 Study site

The experiment is located in hillock and dry land belonging to the Yuzhou experimental base of water conservation agriculture, Henan Academy of Agricultural Sciences. The annual precipitation is 674 mm, of which more than 60% occurs during the summer, and serious droughts typically occur mostly during the spring, but also sometimes in the summer and autumn. The soil parent material is loess material, the soil organic matter is 14.2 g/kg, the total nitrogen is 0.87 g/kg, the hydrolyzed nitrogen is 87.2 mg/kg, the available phosphorus is 12.4 mg/kg and the available potassium is 124.3 mg/kg in the topsoil. The agricultural system is mainly a wheat and corn double cropping system.

### 2.2 Experimental design

The experimentally applied SAP is a nutritional and drought resistant water absorbent material independently developed by the Henan Academy of Agricultural Sciences. The main components include polyacrylamide substances, rare earth, trace elements, nitrogen, phosphorus and potassium by weight dosage ratio of 70-80: 0.1-0.9: 0.5-1.5: 2-8: 2-8: 2-6. Its water absorption ratio is 168. Four treatments were set up in the experiment: (1) no SAP (CK), (2) SAP, 15 kg ha^-1^ (L), (3) SAP, 30 kg ha^-1^ (M), (4) SAP, 45 kg ha^-1^ (H). Each treatment is replicated three times and arranged randomly in the field. The area of each plot was 30 m^2^ (5 × 6 m). Water and fertilizer management, pest and weed management, and other farmland management measures are consistent for all treatments. The rate of nitrogen, phosphorus and potassium application is 210, 90, 90kg ha^-1^, respectively. The proportion of nitrogen fertilizer applied as base fertilizer is 70%, and the other 30% was applied after the jointing stage of wheat. The phosphorus and potassium fertilizer is applied once while sowing, and the SAP is applied in strips along with the bottom fertilizer. According to precipitation and soil moisture, irrigation (30 mm) (Sprinkler irrigation system) was employed during the regreening stage of winter wheat in 2011, 2012, 2013, 2014, 2015, 2017, 2018 and the irrigation was performed at 45 and 60 mm in 2016 and 2019, respectively. The winter wheat (*Triticum aestivum L.*) cultivar Aikang-58 was used from 2011 to 2019.

### 2.3 Measurement of wheat photosynthetic parameters

Net photosynthesis rate (Pn) and transpiration rate (Tr) at filling stages of winter wheat was measured at 9:30–11:00 AM of sunny and windless days from 2015 to 2019. The measurements were made using a Li-6400 portable photosynthesis system (Li-Cor Company, Lincoln, NE, USA). Nine replicates were determined for each treatment. Leaf water use efficiency (LWUE) (the amount of assimilated CO_2_ by plant divided by per unit mass of water) was measured with Fischer and Turner (1978) and Powles’s (1984) method and expressed as:


(1) LWUE=Pn/Tr

(LWUE, µmol CO_2_ mmol^−1^ H_2_O; Pn, µmol CO_2_ (m^2^·s)^−1^; Tr, mmol H_2_O (m^2^·s)^−1^)

### 2.4 Measurement of soil moisture and access to meteorological data

Soil moisture was measured with the oven drying method. The precipitation data used was obtained from the Yuzhou meteorological observation station. Soil water consumption and water consumption of wheat is calculated from the water balance (Lücke and Johnson, 2009).

#### 2.4.1 Soil water consumption and water consumption of wheat

Soil water consumption and water consumption of wheat were calculated using the water balance equation (Li et al., 2010):


(2) $$C_{Soil}=M_{i}-M_{i+1}$$

(3) $$C_{Wheat}=P+I-D-R+W+C_{Soil}$$

where C_Soil_ is, soil water consumption, the decrease in soil water storage (mm), M*_i_* is soil water in the 100 cm soil layer before wheat sowing (mm), M*_i_*_+1_ is soil water in the 100 cm soil layer after harvest of wheat (mm), C_Wheat_ is water consumption of wheat (mm), P is precipitation (mm) data from weather stations near the field, I is irrigation (mm), D is soil water drainage (mm), R is surface runoff (mm), and W is groundwater recharge in the experimental site (mm). There is no drainage observed in our study area. In addition, there was negligible surface runoff in the experiment, and the water table was more than 10 m deep in our study, so R and W were ignored.

#### 2.4.2 Water use efficiency

Water use efficiency (WUE) was calculated as follows (Faramarzi et al., 2010):


(4) $$WUE=Y/C_{Wheat}$$

where Y is the grain yield (kg/ha) and C_Wheat_ is the water consumption of wheat over the winter wheat growing seasons (mm).

### 2.5 Nitrogen use efficiency

Nitrogen use efficiency was calculated using soil nitrogen balance method (Zhou and Shen, 2013):


(5) $$N_{ue}=P_{N}/S_{N}$$

where N_ue_ is nitrogen use efficiency (%), P_N_ is nitrogen content absorbed by wheat grains and plants (kg ha^-1^), and S_N_ is is the sum of the nitrogen from fertilizer, irrigation, atmospheric sedimentation, and reduction of the nitrogen storage in the 1m soil layer(kg ha^-1^).

Soil total nitrogen is measured with alkaline solution diffusion method (Ma and Li, 1997) and then converted into nitrogen storage in the 1m soil layer. Plant nitrogen is measured with the sulfuric acid-mixed accelerator-distillation method (NY/T2419-2013). The irrigated water contains negligible nitrogen. Values for atmospheric sedimentation of nitrogen are based on Luo et al. (2020).

### 2.6 Yield and production factors of wheat

Plant height and spike length were measured with a ruler, and spikelet number, spike grain number per spikelet and sterile spike were counted manually. Grains were weighed a thousand at a time with an electronic balance. The wheat plant population was counted per one square metre and upscaled into number per hectare. Crop yields were determined by manual harvesting and air-drying the grains (12.5%) from 4 m^2^ areas chosen at random in each plot for winter wheat, then converted into yield (kg) per hectare.

### 2.7 Determination of soil microbial biomass carbon and nitrogen, soil enzyme activity, soil organic carbon and soil aggregates

Determination of soil microbial biomass carbon was performed with the chloroform fumigation extraction method (Vance et al., 1987). 3,5-dinitro salicylic acid was used to determine soil sucrase and cellulase activity (Qin et al., 2020) and soil samples were screened for 1 mm before determination. The soil organic carbon content was determined using a heavy cadmium acid potassium outside heating method (Westerman, 1990) and soil samples were screened for 0.25 mm before determination. The size distribution of water-stable aggregates was determined using the wet sieving method (Elliot, 1986). The aggregated soil was separated into different size fractions by gently shaking the samples into the water through a range of sieves to obtain the aggregate size fractions< 0.25 mm, 0.25-0.5 mm, 0.5-1.0 mm, 1.0~2.0 mm, and >2 mm.

### 2.8 Statistical analysis

**Figure 1:**
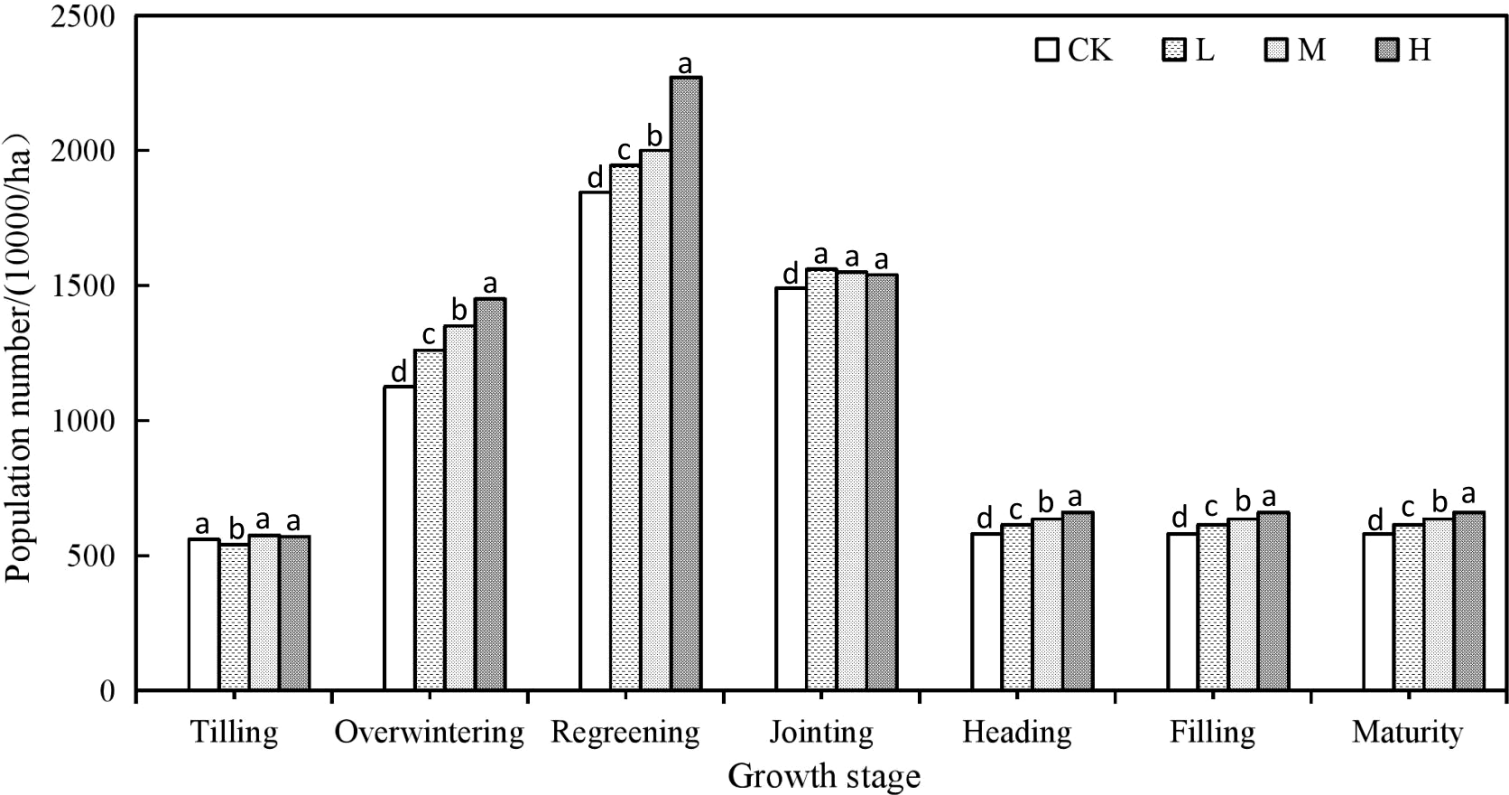
Dynamic characteristics of wheat population number in different growth stages under different rate of suoer absorbent polymer in 2018-2019. CK, 0 kg ha ^-1^; L, 15 kg ha^-1^, M, 30kg ha^-1^; H, 45 kg ha^-1^. Columns labelled with different letters represent significant fifferences between four treatments in the same year by Scheffé test (P<0.05).

**Figure 2:**
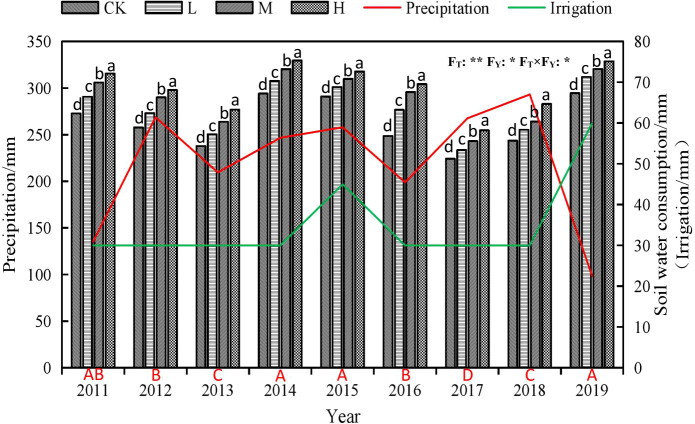
Characteristics of soil water consumption under different rate of super absorbent polymer and precipitation from 2011 to 2019. Notes: CK, 0 kg ha^-1^; L, 15 kg ha^-1^; M, 30 kg ha^-1^; H, 45 kg ha^-1^. Different lower-case letters above columns indicate significant differences between four treatments in the same year by Scheffé test (*P*< 0.05). Different upper-case letters under columns indicate significant differences between mean value of four treatments in different years by Scheffé test (*P*< 0.05). F_T_, F_Y_ and F_T_×F_Y_ mean F-values of treatments, years and their interactions in variance analysis respectively. “* and **” indicate difference at the 0.05 and 0.01 levels, respectively.

**Figure 3:**
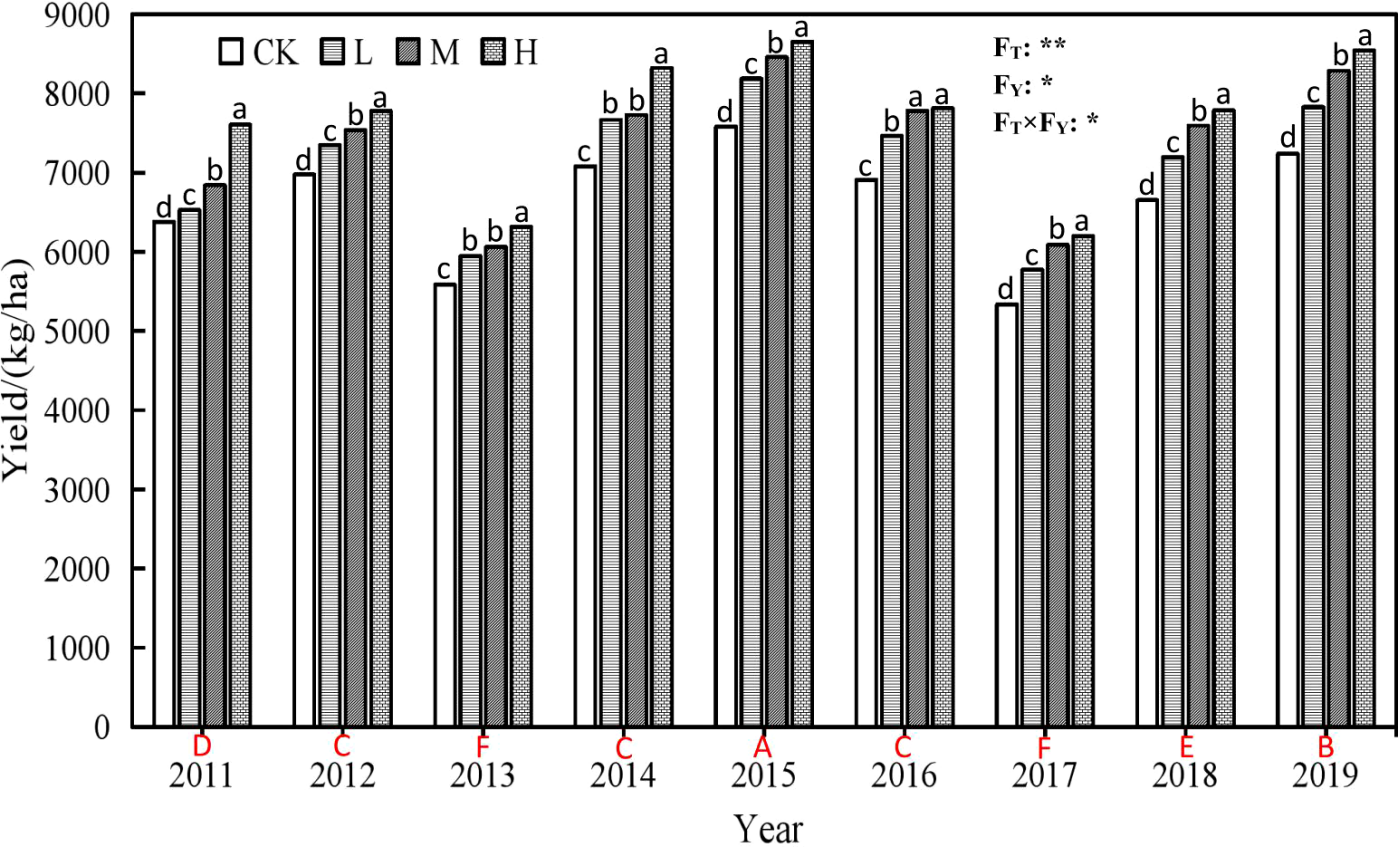
Winter wheat yield under different rate of super absorbentpolymer and precipitation from 2011 to 2019. Notes: CK, 0 kgha-1; L, 15 kg ha-1; M, 30 kg ha-1; H, 45 kg ha-1. Different lowercaseletters above columns indicate significant differencesbetween four treatments in the same year by Scheffé test (P<0.05). Different upper-case letters under columns indicatesignificant differences between mean value of four treatments indifferent years by Scheffé test (P< 0.05). FT, FY and FT×FY meanF-values of treatments, years and their interactions in varianceanalysis respectively. “* and **” indicate difference at the 0.05and 0.01 levels, respectively.

**Figure 4:**
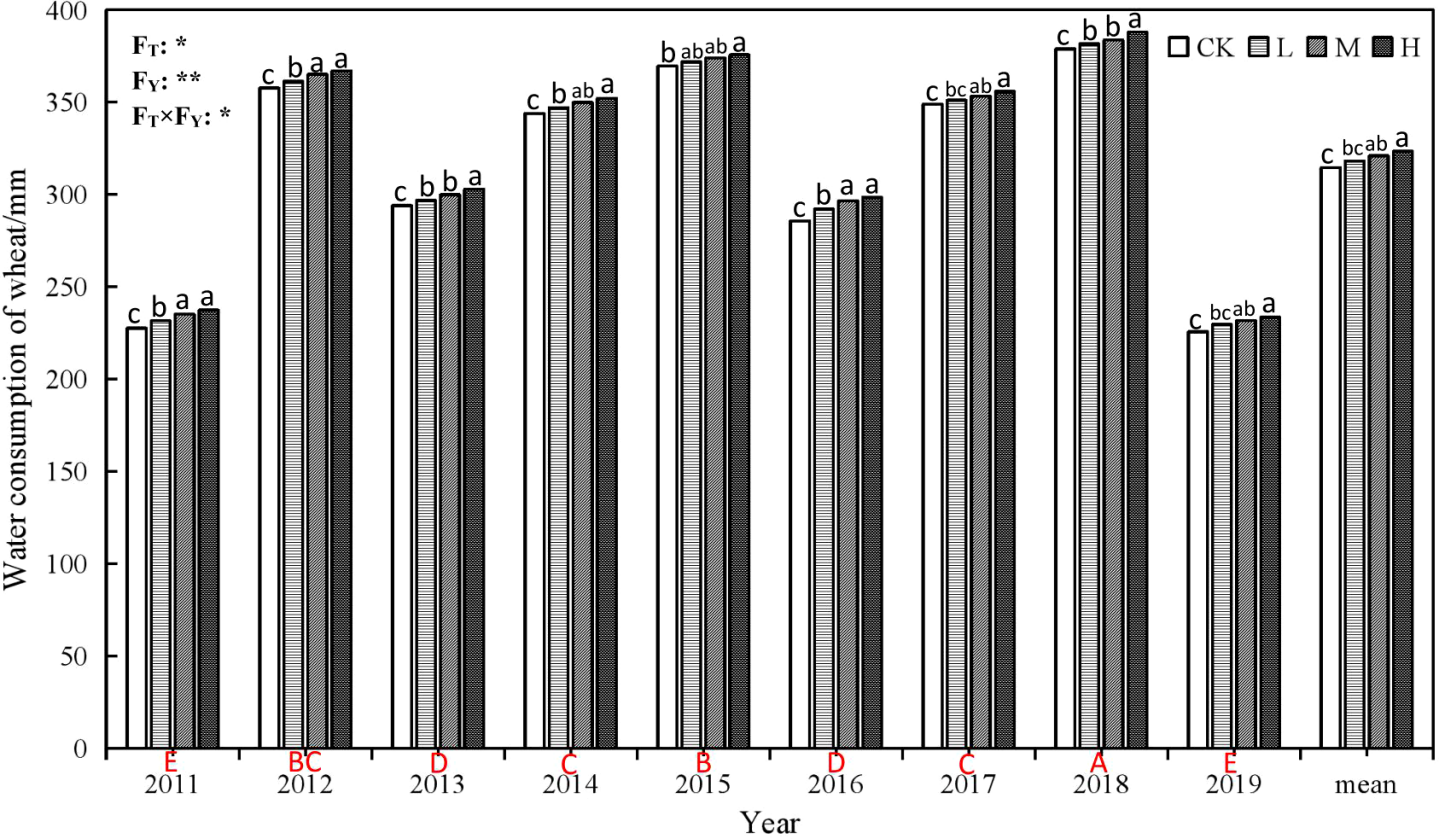
Water consumption of winter wheat under different rate of super absorbent polymer and precipitation from 2011 to 2019. CK, 0 kg ha-1; L, 15 kg ha-1; M, 30 kg ha-1; H, 45 kg ha-1. Different lower-case letters above columns indicate significantdifferences between four treatments in the same year by Scheffétest (P< 0.05). Different upper-case letters under columnsindicate significant differences between mean value of fourtreatments in different years by Scheffé test (P< 0.05). FT, FY andFT×FY mean F-values of treatments, years and their interactionsin variance analysis respectively. “* and **” indicate difference atthe 0.05 and 0.01 levels, respectively.

**Figure 5:**
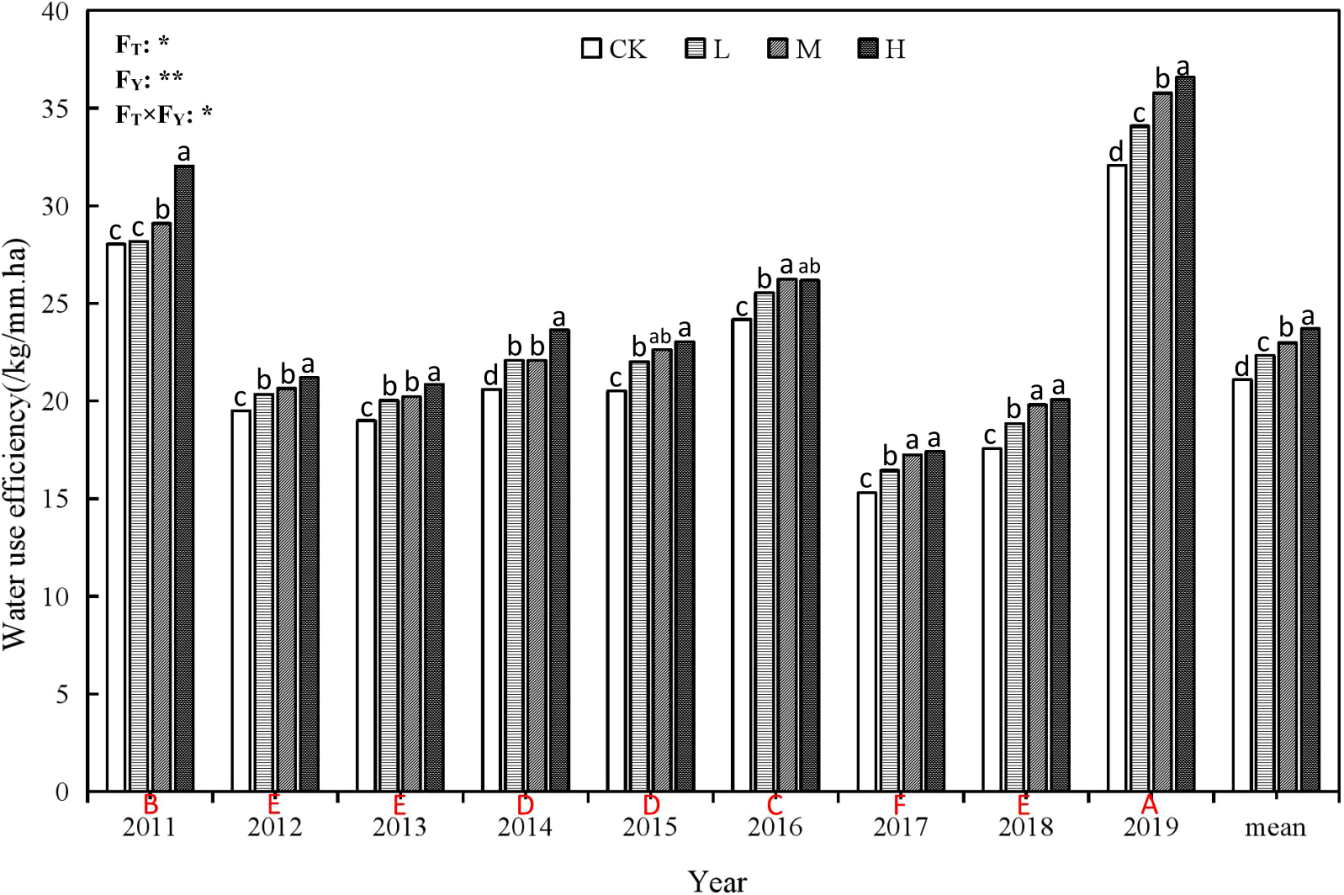
Water use efficiency of winter wheat under different rate ofsuper absorbent polymer and precipitation from 2011 to 2019.CK, 0 kg ha-1; L, 15 kg ha-1; M, 30 kg ha-1; H, 45 kg ha-1.Different lower-case letters above columns indicate significantdifferences between four treatments in the same year by Scheffétest (P< 0.05). Different upper-case letters under columnsindicate significant differences between mean value of fourtreatments in different years by Scheffé test (P< 0.05). FT, FY andFT×FY mean F-values of treatments, years and their interactionsin variance analysis respectively. “* and **” indicate difference atthe 0.05 and 0.01 levels, respectively.

**Figure 6:**
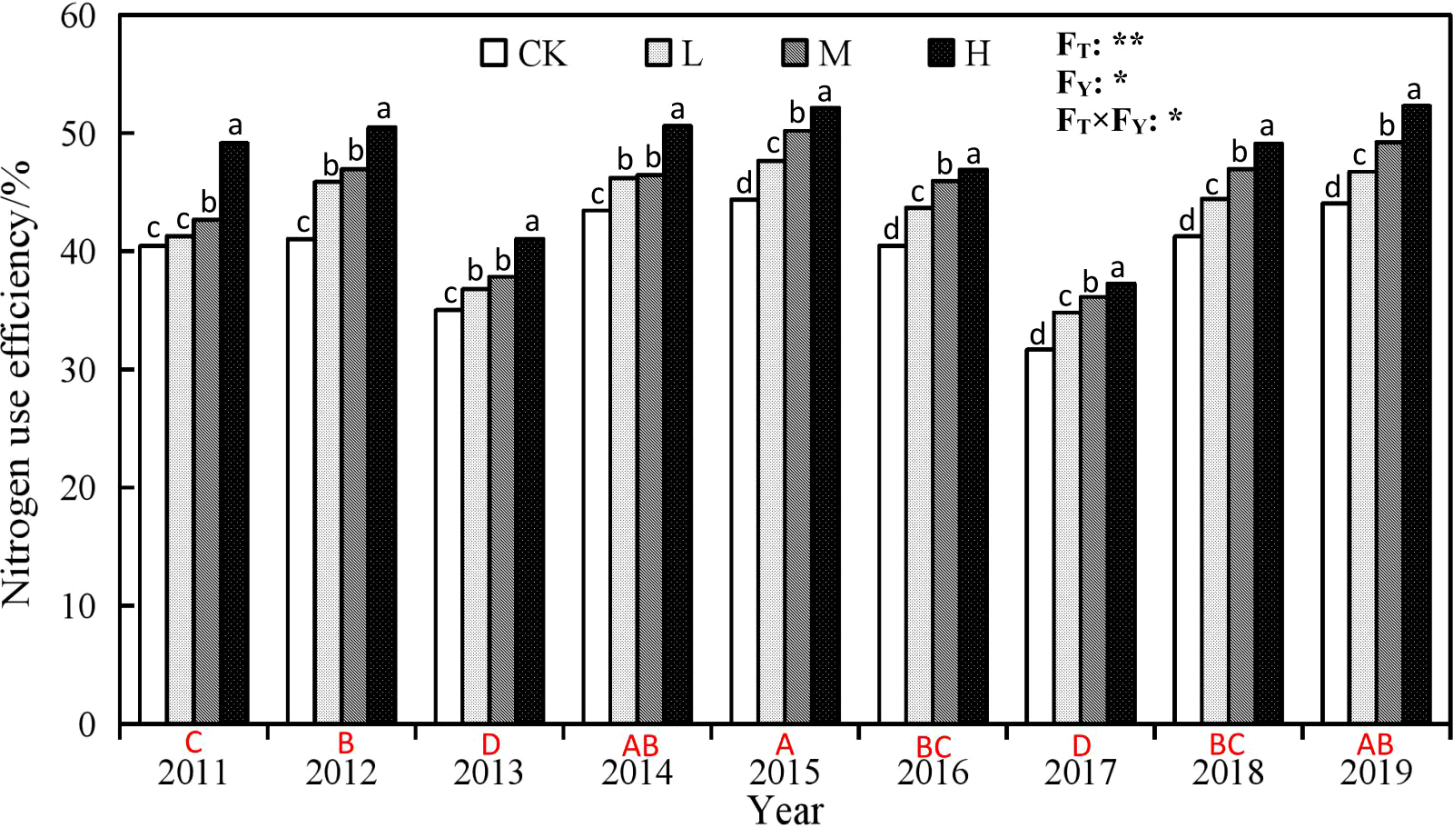
Nitrogen use efficiency of winter wheat under different rate ofsuper absorbent polymer and precipitation from 2011 to 2019.CK, 0 kg ha-1; L, 15 kg ha-1; M, 30 kg ha-1; H, 45 kg ha-1.Different lower-case letters above columns indicate significantdifferences between four treatments in the same year by Scheffétest (P< 0.05). Different upper-case letters under columnsindicate significant differences between mean value of fourtreatments in different years by Scheffé test (P< 0.05). FT, FY andFT×FY mean F-values of treatments, years and their interactionsin variance analysis respectively. “* and **” indicate difference atthe 0.05 and 0.01 levels, respectively.

All reported values were means of the three replicates of each treatment, except for the photosynthetic parameters, which were means of the nine replicates of each treatment. Differences in different diameters of aggregates, soil organic carbon, soil microbial biomass carbon, sucrase and cellulase activity, plant height, spike length, spikelet number, spike grain number, sterile spike and 1000 grains weight of wheat and dynamic characteristics of wheat population number between treatments were analyzed using one-way ANOVA, with a least significant difference (Scheffé) (at *P*< 0.05) test (**Tables 1**–**3** and **Figure 1**). Differences in photosynthetic physiological characteristics, soil water consumption, yield, water consumption of winter wheat, water use efficiency and nitrogen use efficiency of winter wheat between treatments and years were analyzed using two-way ANOVA, with a least significant difference (Scheffé) (at *P*< 0.05) test (**Table 4** and **Figures 2**–**6**). The Pearson correlation coefficient was calculated to assess the relationships among photosynthetic rate, transpiration rate, leaf water use efficiency, yield, water and nitrogen use efficiency, as in **Table 5**. The Pearson correlation coefficient was calculated to assess the relationships among soil organic carbon, soil microbial biomass carbon, soil enzyme activity, soil water consumption, water consumption of wheat, soil aggregates, yield, water use efficiency and nitrogen use efficiency, as in **Table 6**. All statistical analyses were performed using SPSS version 19.0 (SPSS Inc., USA) (Xue, 2019).

**Table 1:** The proportion of different diameters of aggregates under different treatments in different years.

Treatments	>2.0mm		1.0-2.0mm		0.5-1.0mm		0.25-0.5mm		<0.25mm	
	2011	2019	2011	2019	2011	2019	2011	2019	2011	2019
CK	19.1dB	21.2dA	8.3abA	8.1bA	10.9bA	10.3bA	18.7bA	17.9bA	43.6aA	41.9aB
L	21.4cB	24.4cA	8.9aB	9.7aA	13.7aA	11.8aB	18.0bB	19.2aA	38.0bA	35.0bB
M	23.9bB	27.3bA	7.4bB	10.2aA	11.7bA	12.0aA	17.7bA	18.5abA	39.3bA	32.0cB
H	25.4aB	30.0aA	8.4abB	9.7aA	14.7aA	12.1aB	20.6aA	19.1aB	30.9cA	29.1dA

CK, no water absorbent; L, 15 kg ha^-1^; M, 30 kg ha^-1^; H, 45 kg ha^-1^. Different lowercase letters within a column mean significant difference between treatments and different uppercase letters within a line indicate significant differences between different year in same treatment by Scheffé test ( P< 0.05).

**Table 2:** Soil organic carbon, soil microbial biomass carbon, sucrase activity and cellulase activity at filling stage of winter wheat under different treatments in different years.

Treatments	Soil organic carbon		Soil microbial biomass carbon (mg.kg^-1^)		Sucrase activity (mg.g^-1^.24h^-1^)		Cellulase activity (mg.g^-1^.72h^-1^)	
	2011	2019	2011	2019	2011	2019	2011	2019
CK	8.28bB	9.41cA	241.10cB	278.26dA	68.10cA	69.53cA	225.94cB	227.21dA
L	9.10a B	11.90bA	309.83bB	322.40cA	75.47bB	79.84b	235.54bB	239.32cA
M	9.40aB	12.30abA	313.12bB	351.21bA	76.72bB	85.86a	237.90bB	245.97bA
H	9.30aB	13.70aA	325.45aB	368.13aA	80.95aB	87.15a	252.74aB	260.57aA

CK, no water absorbent; L, 15 kg ha-1; M, 30 kg ha-1; H, 45 kg ha-1. Different lower-case letters within a column mean significant difference between treatments and different upper-case letters within a line indicate significant differences between different year in same treatment by Scheffé test (P< 0.05).

**Table 3:** Differences in plant height, spike length, spikelet number, spike grain number, sterile spike and 1000 grains weight of wheat under different treatments in 2019.

Treatments	Plant height(cm)	Spike length(cm)	Spikelet number(spike)	Spike grain number(grain)	Sterile spike(spike)	1000 grains weight(g)
CK	58.2c	6.6a	17.1c	28.7c	3.8a	32.3c
L	60.5b	6.7a	17.8ab	31.4b	2.2c	34.5b
M	60.0b	6.8a	17.4bc	30.6b	3.3ab	36.3a
H	63.4a	6.9a	18.2a	34.4a	3.2b	36.8a

CK, no water absorbent; L, 15 kg ha^-1^; M, 30 kg ha^-1^; H, 45 kg ha^-1^. Different lowercase letters within a column mean significant difference between treatments by Scheffé test (P< 0.05).

**Table 4:** Photosynthetic physiological characteristics at filling stage of winter wheat under different treatments in different years.

Factors	Treatments	2015	2016	2017	2018	2019	Two-Way-ANOVA
Pn (µmol(CO_2_)m^-2^s^-1^)	CK	14.03cB	9.03cC	5.04cE	11.42cA	7.89cD	F_T_: ^*^ F_Y_: ^**^ F_T_×F_Y_: ^**^
	L	15.05bA	10.95bC	6.00bD	13.45bB	10.19bC	
	M	15.69bA	11.19bC	6.87abD	13.71bB	10.62bC	
	H	17.01aA	14.13aC	7.74aE	16.01aB	11.33aD	
Tr (mmol(H_2_O)m^-2^s^-1^)	CK	3.91bA	1.68aC	1.21dD	3.07cB	3.60cA	F_T_: ^*^ F_Y_: ^**^ F_T_×F_Y_: ^*^
	L	4.06aA	1.21bC	1.43bC	3.59bB	3.81abAB	
	M	3.69bA	1.30bB	1.54aB	3.63bA	3.99aA	
	H	3.69bA	1.04cB	1.30cB	4.01aA	3.70bcA	
LWUE (µmol(CO_2_)mmol^-1^(H_2_O))	CK	3.59bC	5.38dA	4.17cB	3.72bC	2.19cD	F_T_: ^*^ F_Y_: ^**^ F_T_×F_Y_: ^*^
	L	3.71bC	9.03bA	4.18cB	3.75bC	2.67bD	
	M	4.25aB	8.59cA	4.45bB	3.78bC	2.66bD	
	H	4.61aC	13.60aA	5.95aB	4.00aC	3.06aD	

CK, no water absorbent; L, 15 kg ha^-1^; M, 30 kg ha^-1^; H, 45 kg ha^-1^. Pn, Photosynthesis rate; Tr, transpiration rate; LWUE, Leaf water use efficiency. Different lower-case letters within a column mean significant difference between treatments and different upper-case letters within a line indicate significant differences between different year in same treatment by Scheffé test (P< 0.05). F_T_, F_Y_ and F_T_×F_Y_ mean F-values of treatments, years and their interactions in variance analysis respectively. “* and **” indicate difference at the 0.05 and 0.01 levels, respectively.

**Table 5:** Relationships among photosynthetic rate, transpiration rate, leaf water utilization efficiency, yield, water and nitrogen use efficiency.

Index	Pn	Tr	LWUE	Yield	N%	WUE
Pn	1					
Tr	0.590**	1				
LWUE	0.109	-0.679**	1			
Yield	0.801**	0.635**	0.015	1		
N%	0.820**	0.678**	-0.024	0.985**	1	
WUE	0.088	0.341	-0.067	0.637**	0.611**	1

Pn, Photosynthesis rate; Tr, transpiration rate; LWUE, Leaf water use efficiency; WUE, water use efficiency. N%, nitrogen use efficiency. * means significant correlation at 0.05 level, and ** means extremely significant correlation at 0.01 level.

## 3 Results

### 3.1 Effect of different rate of SAP on aggregates in 2011 and 2019

As shown in **Table 1**, a large proportion of<0.25 mm aggregate is present, followed by > 2.0 mm, 0.25-0.5 mm, 0.5-1.0 mm and 1.0-2.0 mm. H treatment results in a higher proportion of aggregates > 0.5 mm compared to other treatments, while CK leads to a lower proportion of aggregates<0.25 mm in 2011 and 2019. Compared to the beginning of SAP application (2011) (**Table 1**), the proportion of > 2.0 mm aggregates increases and<0.25 mm aggregates decreases clearly after 9 years (2019).

### 3.2 Effect of different rate of SAP on soil organic carbon, soil microbial biomass carbon, sucrase activity and cellulase activity under the different treatments in 2011 and 2019

**Table 2** shows that applying SAP increases soil organic carbon content, soil microbial biomass carbon, sucrase activity and cellulase activity. With SAP application, soil microbial biomass carbon, sucrase activity and cellulase activity are increased significantly in 2011 and 2019. Compared to 2011, soil organic carbon content, soil microbial biomass carbon, sucrase activity and cellulase activity are higher in 2019. In all, H treatment results in the best outcome across all treatments.

### 3.3 Effect of different rate of SAP on soil water consumption in winter wheat growth stage in different years

**Figure 2** shows that the soil water consumption of the different treatments in the same year is H > M > L > CK. Compared to CK, soil water consumption under H treatment increased by 6.13-9.80 mm, while that of M treatment increased by 4.31-7.57 mm, and that of F treatment increased by 2.14-4.09 mm. Wet years with high precipitation volumes were 2012, 2014, 2015, 2017 and 2018 respectively, whereas 2019 was extremely dry, and 2011 was somewhat dry (**Figure 2**). All other years had average rainfall volumes. Compared to the years with average rainfall, the consumption of stored soil water during dry years is relatively high, such as in 2011 and 2019. On the other hand, soil water consumption did not necessarily decrease during wet years: in 2017 and 2018 it was significantly reduced, and it was slightly reduced in 2012, but significantly increased in 2014 and 2015 (**Figure 2**).

### 3.4 Effect of different rate of SAP on photosynthetic physiological characteristics of winter wheat

It can be seen from **Table 4** that net photosynthetic rate (Pn) was 2015 >2018 > 2016 > 2019 > 2017. With rate of SAP, the Pn increased gradually. In different years, the Pn under H treatment was the highest compared to other treatments.

However, the influence of different rate of SAP on the transpiration rate (Tr) of wheat varied in different years (**Table 4**). The Tr in 2015, 2018 and 2019 was higher that in 2016 and 2017. In 2015, the Tr under L treatment showed the highest compared to other years. While the Tr under M treatment was the highest in 2017 and 2019, the Tr under CK in 2016 and Tr under H in 2018 showed the highest compared to other years.

Due to the effect of different rate of SAP on wheat Pn and Tr was different, leading to the ratio of the two, the leaf water use efficiency (LWUE) was significantly varied (**Table 4**). With the rate of SAP, the LWUE also increased. Among the different rate of SAP, the H treatment (45 kg ha^-1^) had the highest LWUE in different years.

### 3.5 Effect of different rate of SAP on plant height, spike length, spikelet number, spike grain number, sterile spike and 1000 grains weight of wheat

It can be seen that the application of SAP treatments is conducive to improving the growth and development of winter wheat (**Figure 1** and **Table 3**), which can improve the number of unit population, plant height, ear length, spikelet number, ear grain number and grain quality, while reducing the number of sterile ears. The population dynamics (**Figure 1**) during the different growth periods showed a trend of gradually increasing from the sowing to regreening stage, and is highest at the regreening stage, after which it then gradually decreased, until becoming stable after the heading stage. The number of ears at maturity were 6.16-6.60 million ears per hectare, which is an increase of 0.35-0.80 million ears per hectare compared with the CK (**Figure 1**).

Compared with CK, the plant height of winter wheat increased by 1.8 cm to 5.2 cm, the spike length increased by 0.1 cm to 0.3 cm, the spikelet number increased by 0.3 to 1.1 spikes, the grain number per spike increased by 1.9 to 5.7 spikes, and the 1000-grain weight increased by 2.2 g to 4.5 g, under H treatment which produced the best outcomes. The number of sterile spikes decreased by 0.5 to 1.6 spikes, under L treatment which showed the largest decrease (**Table 3**).

### 3.6 Effect of different rate of SAP on winter wheat yield in different years

**Figure 3** shows that the annual yield oscillated after the initial experimental year (2011). The yield of winter wheat is increased in 2012, 2014, 2015, 2016, 2018 and 2019 respectively compared with 2011, and the better years are 2014, 2015 and 2019 especially for H treatment, which showed increases of 9.42%, 13.79%, and 12.33% respectively compared with H treatment in 2011.

Over the years, compared with the CK, the yield under SAP treatments increased by 2.31% to 19.20%, of which the H treatment led to the highest increase of 15.52%, followed by M (11.12%), and L (7.03%).

### 3.7 Effect of different rate of SAP on water and nitrogen use efficiency

The application of SAP can significantly improve the water consumption (**Figure 4**) and water use efficiency (WUE) of wheat (**Figure 5**). H treatment resulted in the greatest increase of WUE, followed by M and L treatments, compared to CK. Like the yields, the WUE also oscillated over the years, and reflects the changes in soil water consumption (**Figure 2**), while running in the opposite direction to changes in precipitation (**Figure 2**) and water consumption of wheat (**Figure 4**). That is, the WUE is higher in the dryer years, while the water consumption of winter wheat is relatively large in wet years, leading to relatively low WUE.

Compared to CK, the WUE of H treatment increased by 1.70 kg ha^-1^ mm^-1^ to 4.51 kg ha^-1^ mm^-1^, followed by M treatment, which increased WUE by 1.06 kg ha^-1^ mm^-1^ to 3.71 kg ha^-1^ mm^-1^, and then L treatment, which increased WUE by 0.4 kg ha^-1^ mm^-1^ to 2.02 kg ha^-1^ mm^-1^. The year with the largest increase in WUE was 2019 (**Figure 5**). Compared with the initial experimental year (2011a), WUE in 2019 increased (4.03 to 6.68 kg ha^-1^ mm^-1^) and the increase from largest to smallest occurred under treatments H > M > L > CK. The WUE in the other years decreased by 2.62 to 14.62 kg ha^-1^ mm^-1^ and the decrease from largest to smallest occurred in 2017 > 2018 > 2013 > 2012 > 2015 > 2014 > 2016.

The application of SAP also plays a positive role on nitrogen use (**Figure 6**), with H > M > L > CK from largest to smallest, and follows the same trend over the years as the winter wheat yield. The application of SAP increased the nitrogen use efficiency of winter wheat. Compared with the CK, the nitrogen use efficiency of H treatment increased by 5.60 to 9.45%, and the largest increase occurred in 2012. M treatment led increased the nitrogen use efficiency by 2.23% to 5.89%, with the largest increase in 2012, while L treatment increased the nitrogen use efficiency by 0.84 to 4.82%, with the largest increase still in 2012.

Compared to the initial experimental year (2011a), there are 6 years with an increase and 2 years with a decrease in nitrogen use efficiency. Among the 6 years (2012, 2014, 2015, 2016, 2018, 2019) with an increase, the nitrogen use efficiency of winter wheat increased by 0.80% to 7.51%: the CK treatment increased it by 0.80% to 3.90%, the L treatment increased it by 2.38% to 6.36%, the M treatment increased it by 3.28% to 7.51%, with a best year in 2015, while the H treatment increased it by 1.34% to 3.15%, with a best year was 2019. However, the nitrogen use efficiency of winter wheat decreased by 4.51 to 8.08% and 6.47 to 11.86% respectively in 2013 and 2017 compared with that in 2011.

### 3.8 Correlation analysis of net photosynthetic rate, transpiration rate, leaf water use efficiency, yield, water and nitrogen use efficiency

From **Table 5**, Pn shows extremely significant (*P*<0.01) positive correlations with Tr, yield and nitrogen use efficiency. Tr shows extremely significant (*P*<0.01) positive correlations with yield and nitrogen use efficiency, but extremely significant (*P*<0.01) negative correlations with LWUE. In addition, nitrogen use efficiency shows extremely significant (*P*<0.01) positive correlations with WUE and yield.

### 3.9 Correlation analysis of soil organic carbon, soil microbial biomass carbon, soil enzyme activity, soil water consumption, water consumption of wheat, soil aggregates, yield, water use efficiency and nitrogen use efficiency

From **Table 6**, Soil organic carbon and soil microbial biomass carbon show significant (*P*<0.05) or extremely significant (*P*<0.01) positive correlations with sucrase activity, cellulase activity, soil water consumption and the proportion of > 2.0 mm and 1.0-2.0 mm aggregates. In addition, soil organic carbon, soil microbial biomass carbon, sucrase and cellulase activities, soil water consumption, and the proportion of > 2.0 mm and 1.0-2.0 mm aggregates show significant (*P*<0.05) or extremely significant (*P*<0.01) positive correlations with yield, water use efficiency and nitrogen use efficiency. However, soil organic carbon, soil microbial biomass carbon, yield, water use efficiency and nitrogen use efficiency show significant (*P*<0.05) or extremely significant (*P*<0.01) negative correlations with the proportion of<0.25 mm aggregates.

**Table 6:** Relationships among soil organic carbon, soil microbial biomass carbon, soil enzyme activity, soil water consumption, water consumption of wheat, soil aggregates, yield, water and nitrogen use efficiency.

Index	Soil organic carbon	Soil microbial biomass carbon	Sucrase activity	Cellulase activity	Soil water consumption	Water consumption of wheat	Proportion of soil aggregates				
							>2.0mm	1.0-2.0 mm	0.5-1.0mm	0.25-0.5mm	<0.25mm
Soil organic carbon	1	0.826*	0.838**	0.708*	0.821*	0.151	0.873**	0.805*	0.002	0.182	-0.752*
Soil microbial biomass carbon	0.826*	1	0.969**	0.892**	0.948**	0.607	0.948**	0.621	0.446	0.277	-0.911**
Yield	0.916**	0.831*	0.858**	0.787*	0.910**	0.261	0.912**	0.726*	0.089	0.416	-0.847**
Water use efficiency	0.920**	0.763*	0.785*	0.690	0.847**	0.111	0.848**	0.753*	-0.037	0.345	-0.766*
Nitrogen use efficiency	0.827*	0.839**	0.876**	0.898**	0.924**	0.440	0.933**	0.628	0.274	0.584	-0.924**

*means significant correlation at 0.05 level, and ** means extremely significant correlation at 0.01 level.

## 4 Discussion

### 4.1 Effect of different application rates of SAP on soil structure, soil organic carbon, soil microbial biomass carbon and soil enzyme activity

SAP acts as a soil amendment, which can improve soil structure, soil pore characteristics (Yang et al., 2011c; Yang et al., 2018) and soil physical properties (Yang et al., 2021; Yang et al., 2022), which in turn increases soil water retention and then facilitates microorganism growth. SAP could be strongly adsorbed to the soil particles, preventing dispersion of them (Ajwa and Trout, 2006). However, the effect of SAP on the soil structure depends on time and environment. When SAP is applied for only a short time, it cannot fully interact with the soil. Therefore, there might have been insufficient time for the SAP to bind with the soil particles. In the first year, part of the SAP would have gradually penetrated into the pores within the aggregates (Lu and Wu, 2003) and preserved or increased soil aggregation and pore continuity (Keren and Ben-Hur, 1997; Ajwa and Trout, 2006). In this study, we found that applying SAP increases the number of > 0.25 mm aggregates and decreased the number of<0.25 mm aggregates one year after application (2011). Gradually, some SAP degrades over time because of sunlight, moisture, and microbial decomposition, etc which then reduces the function of the cemented soil. To overcome the gradual degradation of the SAP and the reduction of its effectiveness, we applied SAP annually before wheat sowing every year. Compared with 2011, the proportion of >2.0, 1.0-2.0 and 0.5-1.0 mm aggregates increased clearly and the proportion of<0.25 mm aggregates decreased with continuous SAP application untill 2019, which facilitates microbial growth (**Tables 1**, **2**), because of positive correlation between >1.0 mm aggregate and soil enzyme activity (**Table 6**).

The soil organic matter (SOM) is considered as the source of energy for diverse soil micro-organisms. In this study, we found that the soil organic carbon is increased with the increase of SAP at the beginning of applying SAP in 2011 and continuous using for 8 years in 2019 and the soil organic carbon in 2019 increase higher than that in 2011. This indicates that SAP can provide sufficient SOM as the food of micro-organisms due to promoting growth of crop root and increment of root exudate and improve microbial activity. Low organic matter availability limits microbial activity that in turn affects crop performance. In addition, the amount of microbial biomass carbon reflects the size of soil microbial activity. We found that SAP also promotes the increment of soil microbial biomass carbon with the increase of dosage of SAP in 2011 and 2019, which indicates SAP stimulates soil microbial activity, especially in 2019 (higher than 2011), which indicates long term application of SAP is conducive to increment of soil microbial biomass carbon and increasing microbial activity.

Activities of soil microbe can be used as a significant indicator of the soil quality in degraded areas (Jeffries and Peter, 2003). Soil microbial activity, closely related to soil enzyme activity, which concerns the cropping system, moisture, and nutrient levels (Moore et al., 2008). The soil enzyme activity may provide valuable information on the main reactions that contribute in slowing decomposition of SOM and nutrients transformation in the soil (Trasar-Cepeda et al., 2000). Soil quality is strongly associated with soil enzymes because of their relationship to soil biology (Dick et al., 2000). Any change in the practices of soil management as well as land use alter the activities of soil enzymes (Ndiaye et al., 2000). In this study, we found that short term (2011) and long term (2019) application of SAP, the scale of 15-45 kg ha^-1^, is beneficial to increases the activity of sucrase and cellulase in the soil, which indicates that SAP increase the growth and capacity of microorganisms in the soil (Li et al., 2014), thereby activating soil nutrients, promoting nutrient absorption by crops and crop generates more substrates under SAP which providing the food and energy for soil microbe (Marschner et al., 2004), which leads to the levels of soil microbial activity and promotes crop growth. The above indicates that sucrase and cellulase were related to soil carbon and soil microbial biomass carbon (shown in **Table 6**).

### 4.2 Effect of different application rates of SAP on photosynthetic physiological characteristics of winter wheat

Under drought conditions, water has an important effect on the photosynthetic properties of wheat (Keck and Boyer, 1974; Tezara et al., 1999; Lawlor and Cornic, 2010), and 90% to 95% of its yield comes from photosynthesis (Li et al., 2006), especially in the late reproductive period, where photosynthates of functional leaves can contribute 80% to the grain (Zheng, 1992). SAP have the function of improving soil structure (Yang et al., 2021; Yang et al., 2022), reducing ineffective soil evaporation (Yang et al., 2009a), and storing water and moisture conservation (Yang et al., 2012). In addition, SAP can regulate the physiological process and improve the photosynthetic efficiency of wheat (Yang et al., 2009b; Yang et al., 2011b) by improving the soil water condition, regulating the relative water content or leaf water potential, and it is of great significance to reduce or slow down the damage degree of crops subject to drought stress.

In this study, we found that the net photosynthetic rate (Pn) is significantly increased along with the increase of SAP dosage although the water supply to the crops varies greatly due to different precipitation. Despite less rainfall in 2019, compared to other years, the H treatment has the largest increase in the Pn, with a 43.6% increase over the CK. These indicate that long-term application of SAP is more beneficial in improving the photosynthetic physiological characteristics of wheat and promoting Pn, which is due to an improvement of soil structure and aggregate stability (Dossou-Yovo et al., 2016; Zhang et al., 2017) and an increase and retention in soil water content (Zhang et al., 2015) and SAP is like a reservoir that absorbs large amounts of water and releases water to the crop in an arid environment, thus promoting normal crop growth and increasing photosynthetic capacity especially in drought year in response to climate change.

However, the influence of SAP on the wheat Tr is not consistent with the effect on the Pn. Except for 2018, the Pn of H treatment was significantly higher, but its Tr was lower. In addition, in 2016 with less rainfall, SAP decreased the Tr significantly, and the Tr under H treatment was the lowest compared to other treatment. However, in 2018 with the more rainfall, with the increase of SAP, the Tr of wheat increased significantly. These indicate that SAP is more conducive to reduce the ineffective transpiration of wheat, promote the use of water.

Therefore, the inconsistency of SAP on Pn and Tr resulted in significant changes in leaf water use efficiency (LWUE). Different rate of SAP increases LWUE of wheat and the H treatment leads to the highest LWUE compared with CK in different years. This indicates that SAP can effectively improve the leaf photosynthetic rate while reducing the leaf transpiration, or be more beneficial for improving the Pn of wheat, and the Tr is also relatively higher, but, compared with CK, the Pn increased more significantly than Tr, thus leading to improved the LWUE. This indicates that the long-term application of SAP has a stable soil structure (Yang et al., 2021; Yang et al., 2022) that regulates soil moisture, nutrients, and microbial enzyme activity (**Table 2**) by buffering against the influence of drought stress (Bodner et al., 2015), which is more beneficial in improving the photosynthetic efficiency per unit water utilization, and promoting dry matter accumulation (Shi et al., 2016; Yang et al., 2021; Yang et al., 2022). Correlation analysis (**Table 5**) showed that the increased photosynthetic rate promoted the improved wheat yield and nitrogen utilization efficiency.

### 4.3 Effect of different rate of SAP on soil water consumption, water and nitrogen use of winter wheat

SAP can increase the soil water-holding capacity (Khadem et al., 2010), decreases soil water loss. And SAP can also reduce the loss of nutrients in soil and adsorb nutrients in soil or fertilizer and slowly release (Khodadadi, 2017), making the supply of nutrients in soil more synchronized with plant demand for nutrients (Song et al., 2018), improve nutrient use rate (Busscher et al., 2009), and reduce the environmental pollution of fertilizer (Shaviv and Mikklelsen, 1993). Therefore, SAP can supply suitable water and nutrient and increase crop yield (Ekebafe et al., 2011).

Precipitation has an important impact on crop growth and water use. Excessive rainfall does not necessarily promote water use for crops. We found that the soil water consumption is increased when the precipitation is shortage and the supplement water is insufficient, the soil water consumption is reduced when the precipitation is abundant and the air humidity is high, and the appropriate supplement water is supplied at the same time. However, regardless of the rainfall and irrigation, soil water consumption increases with the increase of SAP dosage. This maybe SAP stimulates crop growth, which leads to high water consumption, which differs from previous studies (Yang et al., 2010). In addition, we found that from **Figure 2**, as the rainfall or irrigation volume increases, the soil water consumption also increases. Therefore, the water consumption of crops can be regulated by controlling the irrigation.

In addition, we found that SAP improves the population number in different growth stage, plant height, ear length, spikelet number, ear grain number and 1000 grain quality, and reducing the sterile ear, which is beneficial to increment of wheat yield. During the research for 9 years, we found that compared with 2011, the yield of winter wheat was decreased in 2013 and 2017 because of the climate or diseases and pests, which reduced by 16.85% and 18.51% respectively compare with that of 2011. The results showed that the effect of SAP on winter wheat yield was influenced by climate at a certain extent. However, applying SAP still increases wheat yield with the increase of SAP dosage. Compared with the CK, although water consumption of wheat increases with the dosage of SAP, compared with the water consumption of wheat, the yields boost more, thus lead to increment of water use efficiency with dosage of SAP, which shows that SAP prolongs plant survival under water stress conditions (Hüttermann et al., 2009) especially in 2011 and 2019 with less rainfall.

While improving crop water use after application of SAP not only improves water use of crop, but promotes nutrient uptake and boosts crop nutrient use because mixed application of SAP with soil retains a large amount of water and nutrients, especially ammonium ions and potassium ions (Magalhaes et al., 1987), which are released when plant needs (Hutteermann et al., 1999), so SAP as nutrient carriers and regulators reduce fertilizer loss while maintaining normal plant growth (Shainberg et al., 1990; Mikkelsen, 1994). In this study, we found that applying SAP increases the nitrogen use efficiency and with the dosage of it, the nitrogen use efficiency boosts significantly in different years. Compared with other years, the nitrogen use efficiency in 2013 and 2017 are lower. While the nitrogen use efficiency in 2019 reaches to the highest compared with other years. This indicates SAP is a good amendment to regulate nutrient in soil and promote crop growth and nutrient use (Ekebafe et al., 2011; Yang et al., 2013; Khodadadi, 2017) especially in years with less rainfall and irrigation (**Figures 2**, **6**). In addition, high microbial biomass and activity often lead to high nutrient availability (Zaman et al., 1999; Tu et al., 2003) and may in turn lead to high plant productivity (Tu et al., 2006), which could explain the higher wheat yield produced by the SAP relative to the other treatments (**Tables 1**, **2**). In addition, the SAP promoted chlorophyll synthesis (Yang et al., 2011e), and maintained a higher leaf area index and photosynthetic rate (Yang et al., 2009b; Yang et al., 2011b), thus prolonging the functional period and increasing the distribution of dry matter (Yang et al., 2013; Yang et al., 2021), and ultimately resulting in increment of crop yields (Xu et al., 2017). From the correlation analysis (**Table 5**), we found that the yield, water use efficiency and nitrogen utilization rate can be improved simultaneously, while the application of SAP is more conducive to promoting the synergistic improvement of the three. However, excessive SAP can cause damage to crops, causing them to compete for water, eventually reducing crop production. Therefore, the amount of SAP should be optimized.

## 5 Conclusion

Through an analysis of the characteristics of soil water consumption over nine years, we find that the application of SAP can amend the soil structure, soil organic carbon, soil microbial biomass carbon and soil enzyme activity, while increasing soil water, improving the photosynthetic physiological characteristics and yield of wheat, and enhancing the water and nitrogen use efficiency. From a correlation analysis, we conclude that SAP can promote the use of crop water and fertilizer by improving soil structure, increasing soil and microbial organic carbon, soil enzyme activity, Pn and improving the crop water accessibility. Considering the variation in precipitation rates at our experimental site, optimizing the winter wheat yield and water and nitrogen use efficiency is attained with an optimal SAP dosage of 45kg ha^-1^.

## Data availability statement

The raw data supporting the conclusions of this article will be made available by the authors, without undue reservation.

## Author contributions

YY wrote the main manuscript. SZ wrote the part of the manuscript and revised the manuscript. JW revised and gave some advice for the manuscript. CG prepared the figure and table of the manuscript. DL performed most of the experiments. DT edited the language and modified the main manuscript. All authors contributed to the article and approved the submitted version.
